# Association of gut microbiota composition and function with an aged rat model of senile osteoporosis using 16S rRNA and metagenomic sequencing analysis

**DOI:** 10.18632/aging.103293

**Published:** 2020-06-02

**Authors:** Sicong Ma, Jinhong Qin, Yongqiang Hao, Lingjie Fu

**Affiliations:** 1 Shanghai Key Laboratory of Orthopaedic Implants, Department of Orthopaedic Surgery, Shanghai Ninth People’s Hospital, Shanghai Jiao Tong University School of Medicine, Shanghai 200011, China; 2 Department of Microbiology and Immunology, Institutes of Medical Sciences, Shanghai Jiao Tong University School of Medicine, Shanghai 200011, China

**Keywords:** gut microbiota, osteoporosis, 16S rRNA sequencing, metagenomics, aged rats

## Abstract

Recently, more interest has been paid to the association between bone mass and gut microecological dysbiosis. The results of clinical studies comparing gut microbiota (GM) in osteoporosis patients have been inconsistent due to different inclusion and exclusion criteria. To date, the association between the GM and senile osteoporosis remains poorly understood. Here, we utilized an aged rat model (22 months old) of senile osteoporosis to study the association of the composition and function of the GM with osteoporosis by 16S rRNA and metagenomic sequencing. The results showed that there was a significant reduction in alpha diversity and the F/B (*Firmicutes/Bacteroidetes*) ratio in aged rats. At the genus level, the enrichment of *Helicobacter* was potentially related to osteoporosis as a risk factor. Metagenomics results based on two databases indicated that shifts in the GM contribute to senile osteoporosis through metabolic pathways and subsequent immune disorders. In conclusion, our study reveals the association of gut microbiota composition and function with senile osteoporosis in an aged rat model in a brand new way, and variations in the GM might contribute to senile osteoporosis through metabolic pathways.

## INTRODUCTION

Senile osteoporosis is a systemic skeletal disease characterized by reduced bone mass and microstructural destruction of bone tissue with increased bone fragility, which is prone to fracture [1]. The pain, deformity and death caused by osteoporotic fractures seriously affect the health of elderly individuals. With rapid rise in aging societies, the number of these cases is expected to increase significantly in the future. The treatment of primary osteoporosis used to be a long duration of medicine, such as bisphosphonates and estrogen. However, there are several problems during medicine treatment, including poor patient compliance and adverse reactions such as gastrointestinal reactions, nephrotoxicity, mandibular osteonecrosis and even increased tumor risk [2]. Therefore, a novel strategy for the treatment of senile osteoporosis is still an urgent research direction.

The gut microbiota (GM) has demonstrated a major effect on metabolism and the immune system in humans and animals [3]. Due to its diversity and complexity, the GM interacts with organisms through the intestinal mucosa or metabolites directly or indirectly [4]. It is considered to be a whole new organ because its composition and function are constantly changing by age, lifestyle, diet, and disease states [5]. The main components that regulate the balance of microecology are *Firmicutes, Bacteroidetes, Actinobacteria*, and *Proteobacteria* [6]. A delicate balance in the GM microecology is crucial in maintaining intestinal immunity and host homeostasis. Lees et al. demonstrated that the age and local environment drove the composition of the GM and were more deterministically important than the host genotype [7]. However, the association of GM composition and function in senile osteoporosis has not been fully elucidated.

Gut microecological dysbiosis is involved in multiple host metabolic pathways. The GM plays a role in calcium absorption-related proteins to regulate bone metabolism [8]. It can also increase osteoclastogenic cytokines in a T-cell-dependent mechanism and drive bone resorption under inflammatory conditions by producing metabolites [9]. Studies with germ-free and antibiotic-treated animals showed bone mass accumulation by decreasing osteoclastic precursor cell number [9–11]. In addition, several clinical studies compared the GM between healthy people and osteoporotic patients, and the results showed that the composition and function were inconsistent among these studies [12–14]. The possible discrepancies of these studies might be due to the different inclusion and exclusion criteria, mixed genders and different diet and patient disease status.

To date, the association between the GM and senile osteoporosis remains poorly understood. In the current study, we utilized an aged rat model of senile osteoporosis (22-month-old female SD rats) to elucidate the association of the gut microbiota composition and function using 16S rRNA gene and metagenomics high-throughput sequencing. The advantage of using animal models is the homogeneity in the sample group and controllability in diet and lifestyle. Compared with mice, rats have a similar GM structure as humans [15].

## RESULTS

### Alterations in body mass index and bone microarchitecture in senile osteoporotic rats

**Figure 1:**
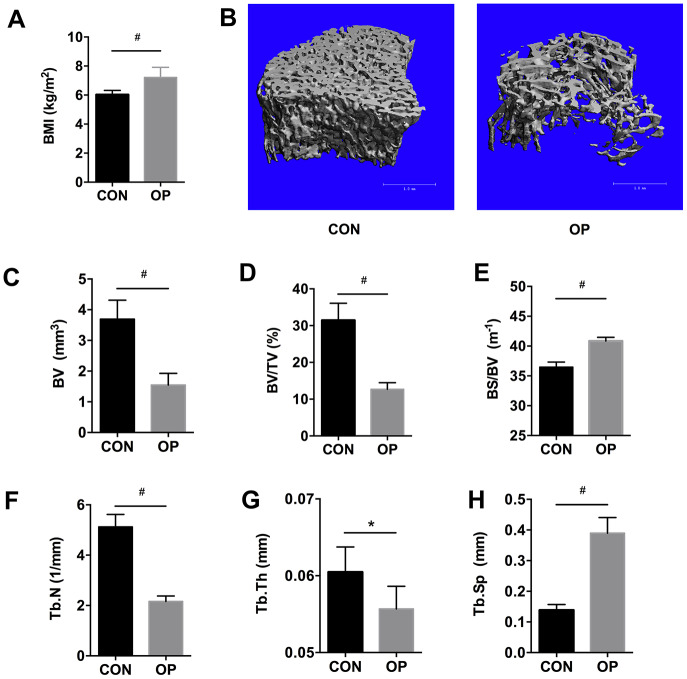
**BMI, micro-CT based three-dimensional images and histomorphometry in aged female SD rats.** BMI (**A**), three-dimensional reconstruction (**B**), bone volume (BV), bone surface density (BV/TV), ratio of bone surface area to bone volume (BS/BV), trabecular number (Tb.N), trabecular thickness (Tb.Th), and trabecular spacing (Tb.Sp) using micro-CT scanning (**C**–**H**). Results were compared by unpaired *t* test with Welch’s correction, *P < 0.05, #P < 0.01.

To verify the aged rat model of senile osteoporosis, micro-CT analysis and body mass index (BMI) were performed. As expected, the BMI was higher in aged rats (Figure 1A, P<0.01). Compared with the control group (the CON group, 6-month-old female SD rats), a deteriorated bone architecture was manifested by the three-dimensional reconstruction of micro-CT in the 22-month-old female SD rats (the OP group) (Figure 1B). Cancellous bone volume (BV) and bone volume fraction (BV/TV) decreased significantly in the OP group (P<0.01, Figure 1C, 1D), and a similar trend was found in trabecular number (Tb.N) and trabecular thickness (Tb.Th) (P<0.05, Figure 1F, 1G). In contrast, bone surface to bone volume (BS/BV) and trabecular separation (Tb.Sp) increased significantly (P<0.01, Figure 1E, 1H). Taken together, these results verified the animal model of senile osteoporosis in aged rats.

### Higher amounts and lower diversity of OTUs in senile osteoporosis

**Figure 2:**
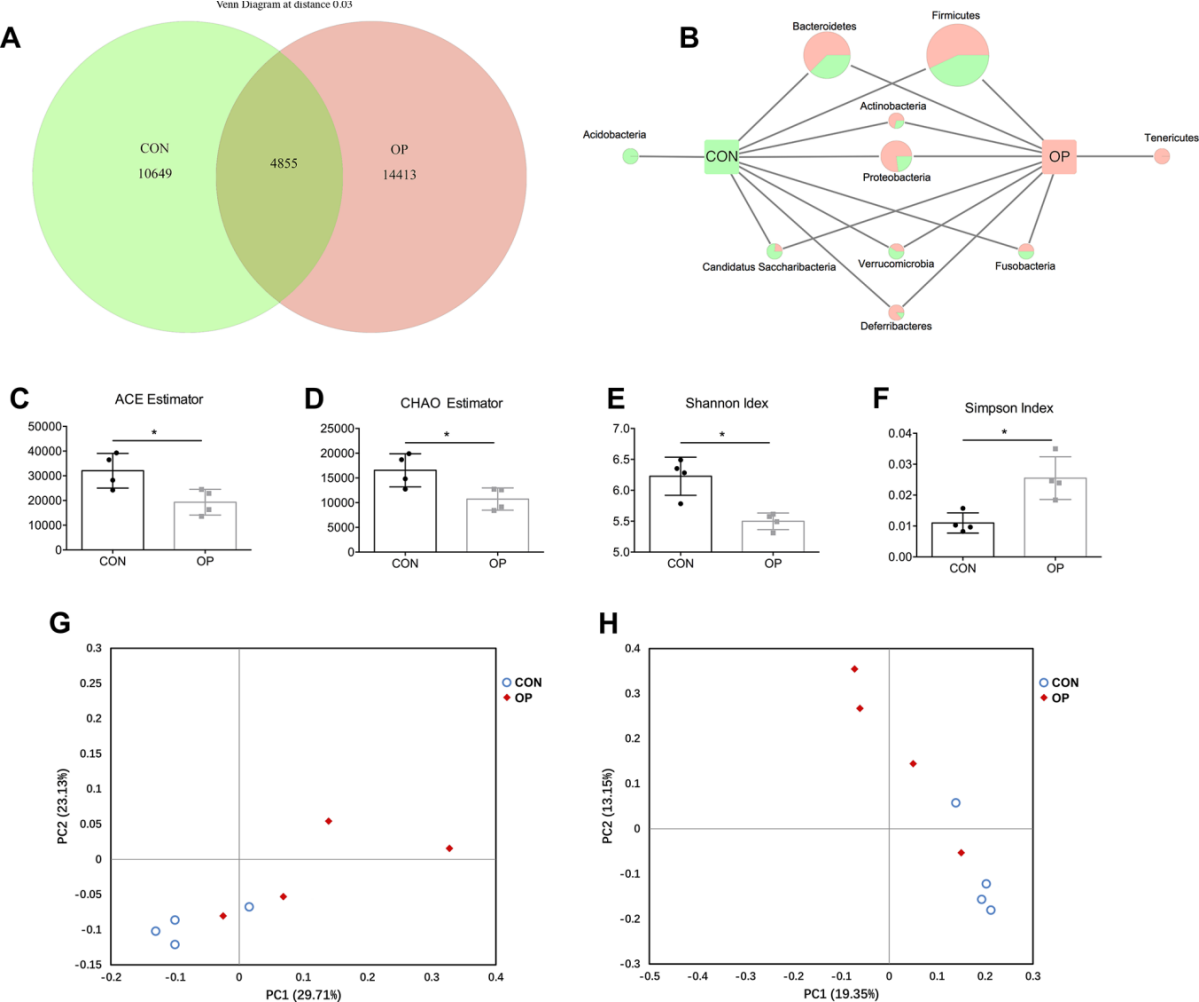
**Venn diagram and microbial community structure assessment in the two groups.** Number of common and specific OTUs for grouped samples with OTUs=0.03 (**A**). Comparison of common and specific microbiota at phylum level (**B**). Community richness assessment contains the ACE estimator (**C**) and the Chao estimator (**D**); community diversity assessment contains the Shannon index (**E**) and the Simpson index (**F**). Principal coordinate analysis of 16S rRNA sequences from all samples using unweight (**G**) and weight (**H**). Results were compared by unpaired *t* test with Welch’s correction, *P < 0.05.

In order to investigate how the gut microbiota is altered in senile osteoporosis, OTUs were compared between the two groups after being clustered at 97% similarity. The total number of OTUs in the CON group and OP group was 15540 and 19268, respectively (Figure 2A). Although 4855 OTUs were common between the two groups, the OP group still had more enriched sequences than the CON group (26.12%). In this case, sequences of *Tenericutes, Bacteroidetes, Actinobacteria, Proteobacteria* and *Deferribacter* were the most abundant in the OP group. For the CON group, *Acidobacteria, Candidatus Saccharibacteria and Verrucomicrobia* were the most abundant. *Fusobacteria* showed no difference between the two groups. In view of the fact that *Firmicutes* is the most abundant microflora, the absolute content is largely related to the number of total OTUs obtained by sequencing, so we determined the difference in the relative contents of *Firmicutes* between the two groups in subsequent analysis (Figure 2B).

Although the number of OTUs was enriched in the OP group, alpha indexes including the ACE estimator, CHAO estimator, Shannon index and Simpson index revealed a lower alpha diversity compared with the control group (P<0.05, Figure 2C–2F). Principal coordinate analysis could also display the microbial structure by using both unweighted and weighted UniFrac. The visualized size of ecological distance between samples showed that the two groups could be divided into two different clusters and separated clearly. Principal coordinate 1 (PC1) (percent variation explained 29.71%) of the unweighted UniFrac separated the CON and OP samples (Figure 2G), while principal coordinate 2 (PC2) (13.15%) of the weighted UniFrac separated samples of two groups (Figure 2H). The diversity analysis revealed that the gut microecological environment was disordered explicitly in senile osteoporosis.

### Microbial components of senile osteoporotic rats were distinct from those of the control rats

**Figure 3:**
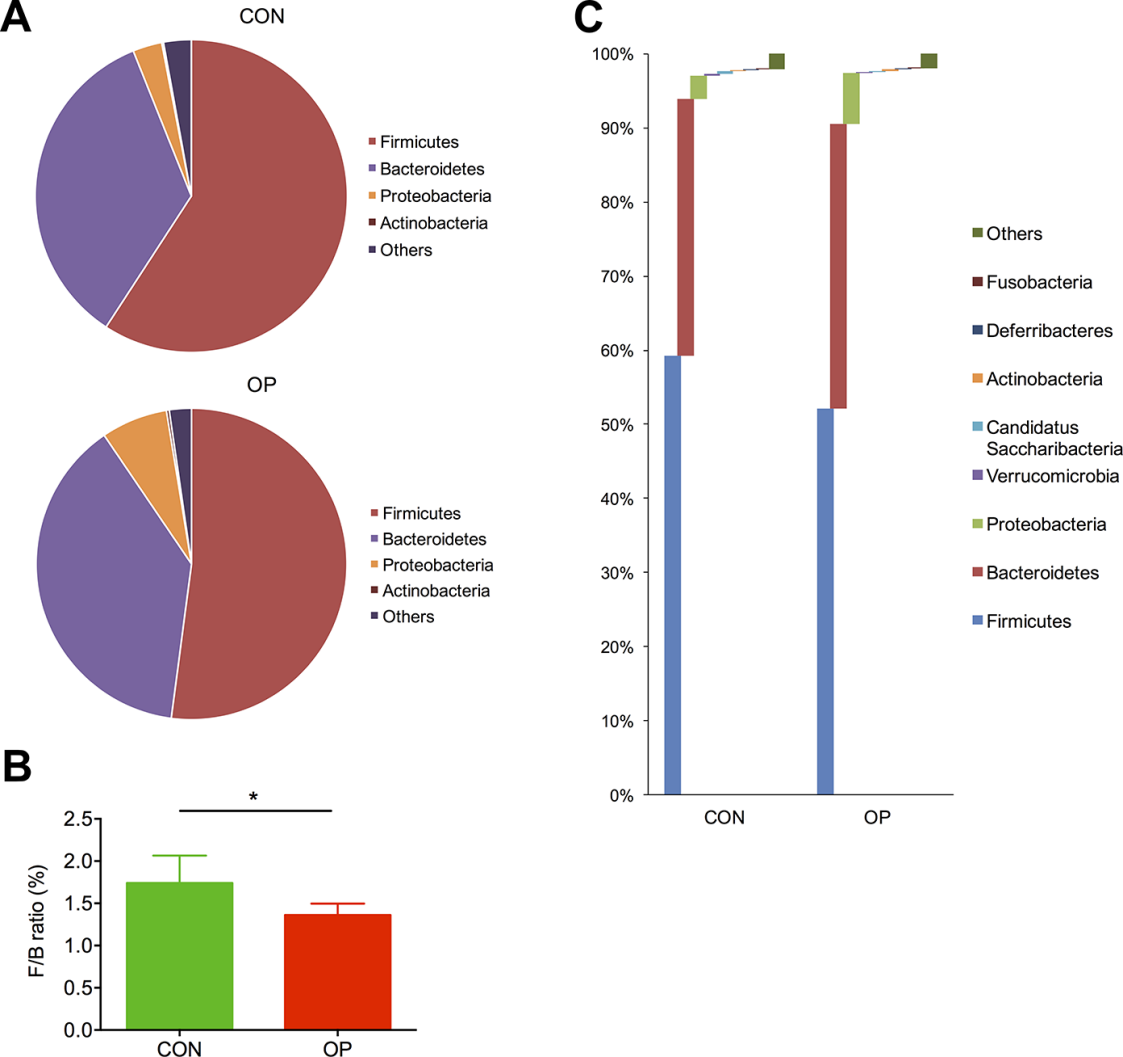
**Differences of taxonomy in phylum level between samples of the control group and senile osteoporosis group.** Annotation of phylum level for the most four abundant species determined by identified 16S rRNA sequences (**A**). F/B ratio difference between both groups (**B**). Comparison of content difference in phylum level for all species (**C**).

Taxonomic differences were further analyzed by using the RDP classifier Bayesian algorithm. At the phylum level, the most abundant bacteria in both groups was *Firmicutes*, which decreased significantly in the OP group, while the other major microbial community, *Bacteroidetes*, showed the opposite tendency (Figure 3A). By calculating the *Firmicutes/Bacteroidetes* ratio (F/B ratio), we identified that there was a great reduction in this value from healthy controls (1.74) to osteoporotic rats (1.36) (P<0.05, Figure 3B). Apart from the relative abundance of those two major phyla, *Proteobacteria* also showed a decreasing trend in the OP group (6.91%, CON: 3.02%, q<0.01) (Figure 3C).

**Figure 4:**
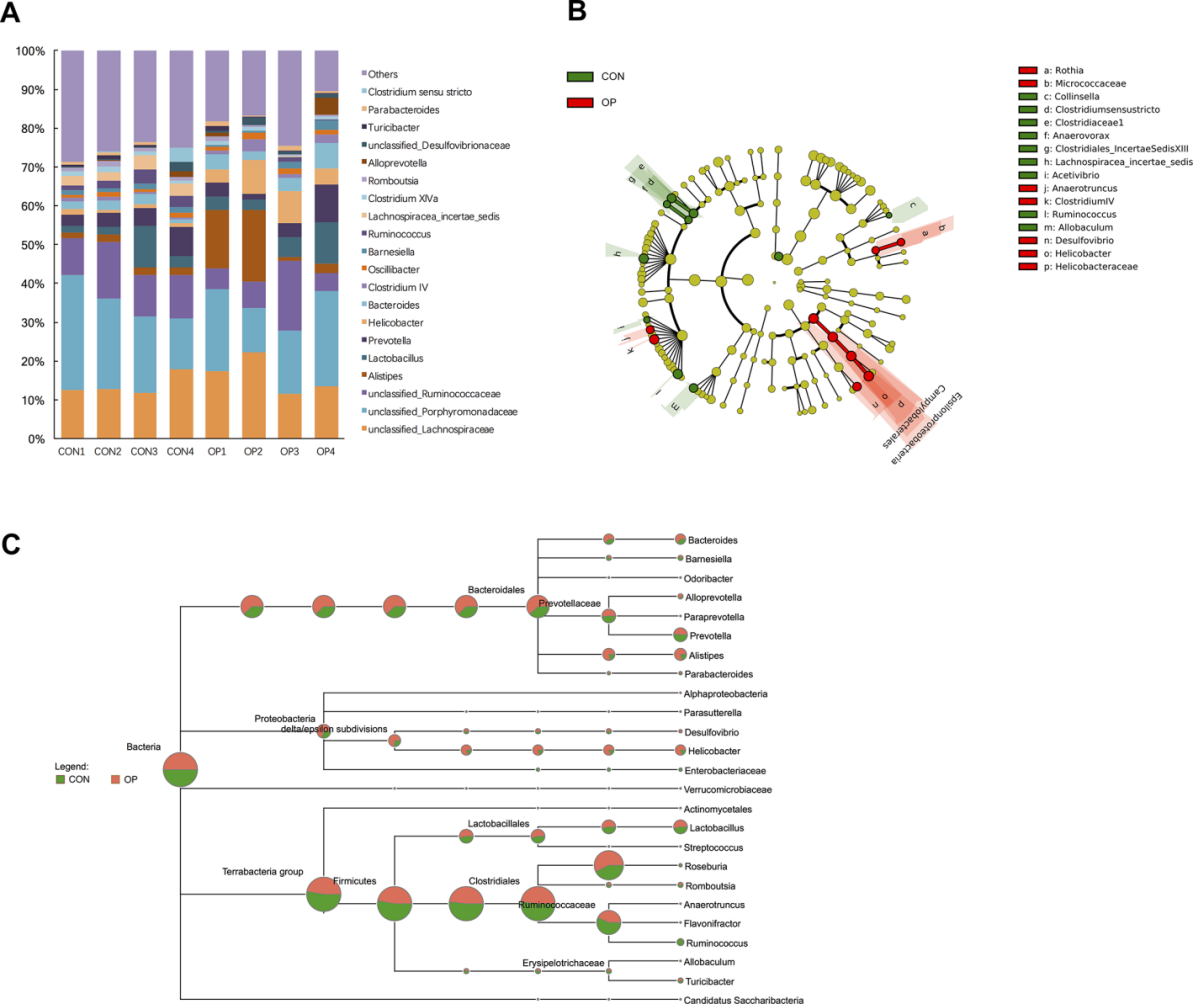
**Differences of taxonomy in genus level between samples of the control group and senile osteoporosis group.** Annotation of phylum level for the most twenty abundant species determined by identified 16S rRNA sequences (**A**). Cladogram of biomarkers for the control group and osteoporosis group (**B**). Map of the differences to taxonomic trees in genus level (**C**).

**Figure 5:**
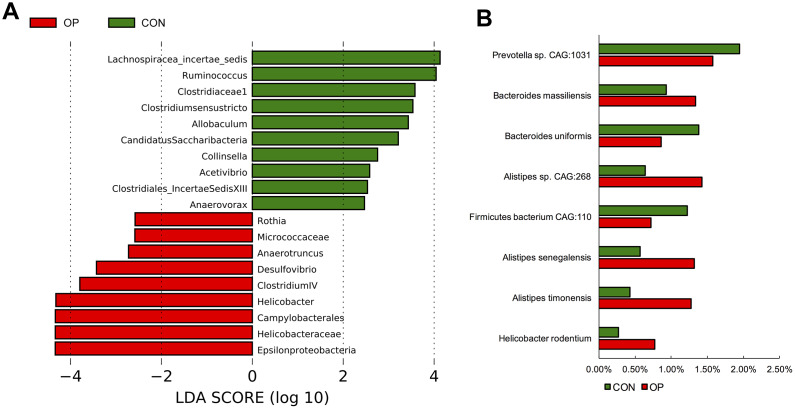
LDA Effect Size at the phylum level of the each group (**A**). The significant differences of species abundance between both groups at species level (**B**).

At the genus level, unclassified_Lachnospiraceae, unclassified_Porphyromonadaceae, unclassified_Ruminicoccaceae, Alistipes, Lactobacillus, Prevotella, and Helicobacter were the most abundant in all samples (Figure 4A). Then, the gut microbiota was analyzed by LDA effect size to determine the significant differences between both groups and classify the bacteria at the corresponding classification level (Figure 4B). The results showed that Clostridium sensu stricto, Clostridiaceae 1, Anaerovorax and Clostridiales_IncertaeSedisXIII (sub-Firmicutes) were more abundant in the CON group, and Helicobacter and Helicobacteraceae (sub-Proteobacteria) were more abundant in the OP group. The majority of the Bacteroidetes phylum (except Prevotella genes) also showed more abundant in the OP group (Figure 4C). Moreover, the four most abundant bacteria related to osteoporosis all belonged to the Proteobacteria phylum and showed a decreasing trend: Epsilonproteobacteria, Helicobacteraceae, Campylobacterales, and Helicobacter. The most negatively correlated bacteria belonged to the Firmicutes phylum: Lachnospiracea_incertae_sedis and Ruminococcus (Figure 5A). In order to identify the precise microbiome associated with senile osteoporosis, we used metagenome sequencing to annotate at the species level (Figure 5B). The presence of Bacteroides massiliensis, Alistipes sp. CAG:268, Alistipes senegalensis, Alistipes timonensis and Helicobacter rodentium might potentially lead to an increase in the phyla Bacteroidetes and Proteobacteria. However, the abundance of Prevotella sp. CAG:1031 and Bacteroides uniformis decreased in the OP group (q<0.01).

**Figure 6:**
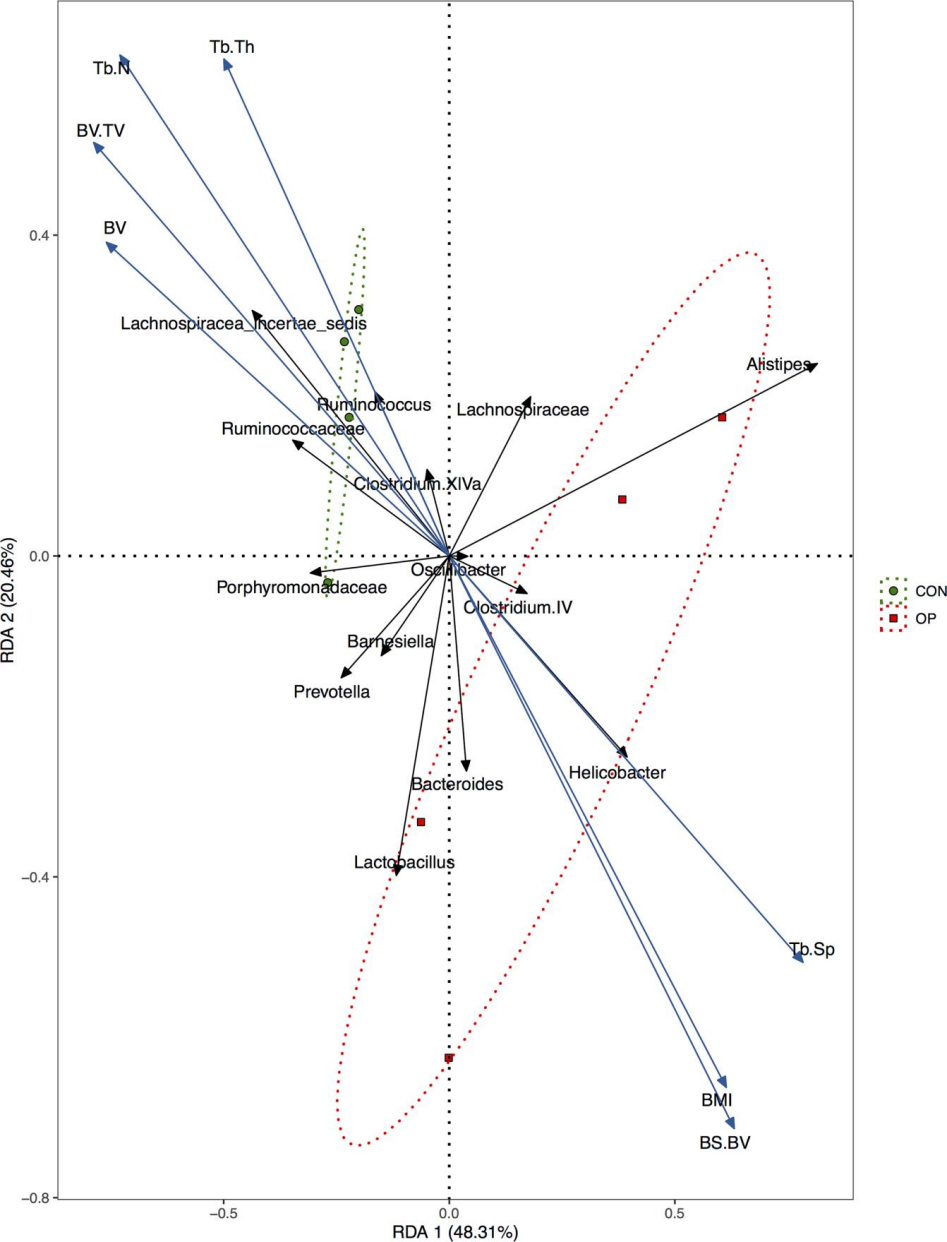
**Redundancy analysis (RDA) of the relationship between BMI, bone histomorphometry parameters, and major bacteria at genus level.**

Redundancy analysis could visualize the association between bone histomorphometry parameters and GM by imaging. The blue arrows represent BMI and each parameter of bone histomorphometry, and the black arrows represent each GM at the genus level. The smaller the angle between two arrows was, the higher the positive correlation was. The green circles and red squares represent the control rat and osteoporotic rat samples, respectively (Figure 6). The results showed that the two groups were obviously separated, which indicated that the taxonomies were significantly different. In addition, *Lachnospiracea incertae sedis*, *Ruminococcus* and *Clostridium XIVa* were closely related to the control group and associated with osteogenesis. Meanwhile, *Helicobacter* and *Bacteroides* were positively correlated with osteoclastogenesis and higher BMI.

### Functional distinction associated with senile osteoporosis due to gut microbiota

**Figure 7:**
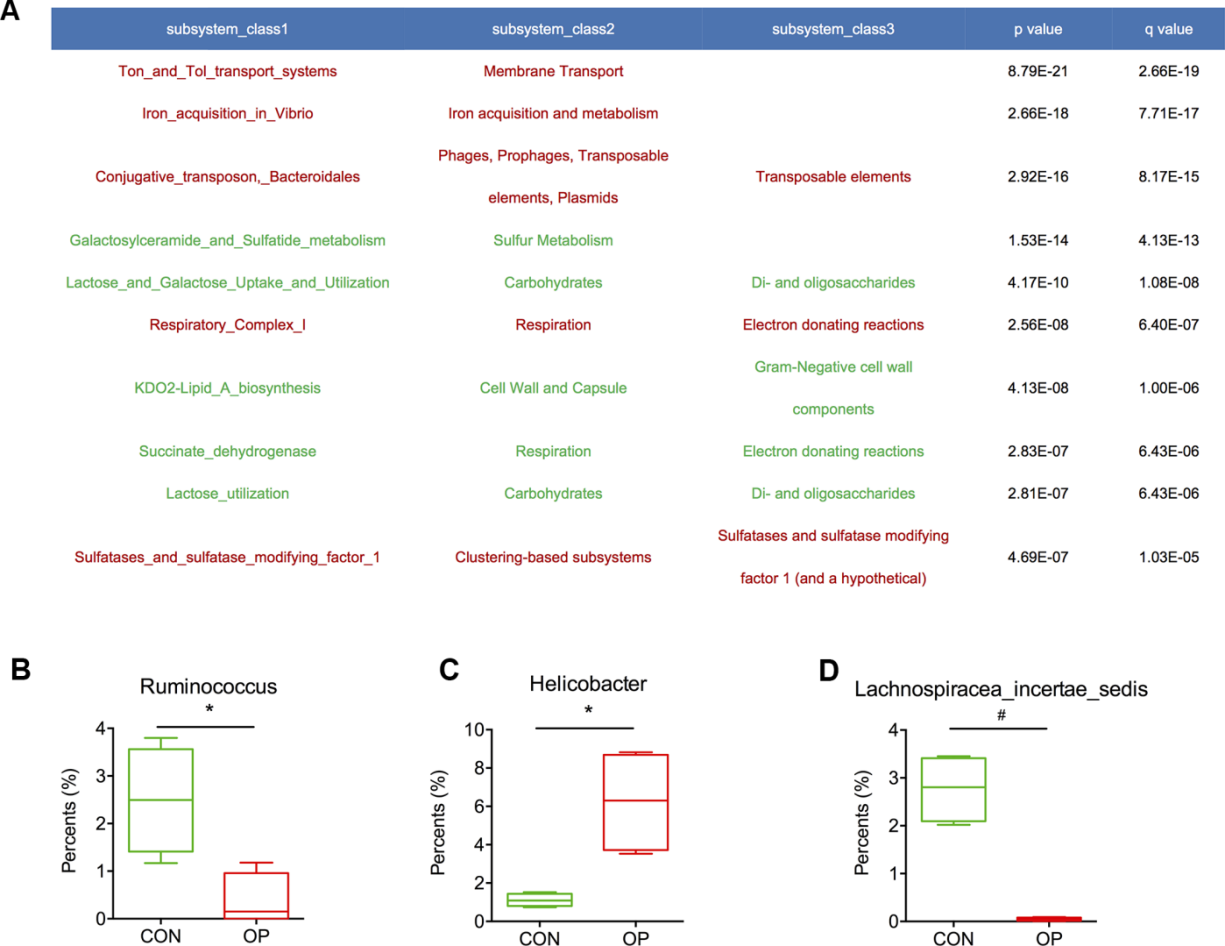
Abundance of functional genes was calculated with significant differences (**A**). The relative proportions of *Ruminococcus*, *Helicobacter* and *Lachnospiracea_incertae_sedis* in the gut microbiota in the control and aged rats (**B**–**D**).

**Figure 8:**
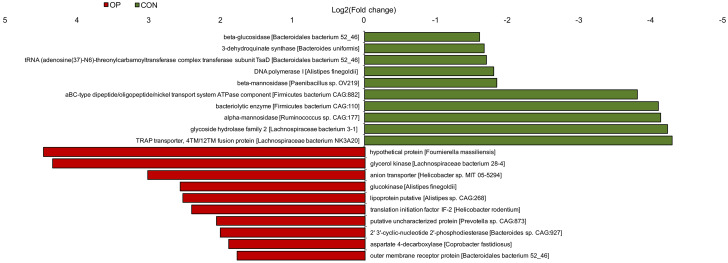
**Histogram of functional gene enrichment analysis (q< 0.01).**

To further determine the functional genes that might be related to senile osteoporosis, metagenome sequencing was performed to analyze significant differences between the CON and OP groups based on the SEED database. Transposable elements, electron donating reactions and sulfatases and sulfatase modifying factor 1 (and a hypothetical) increased significantly in the OP group (q<0.01); in contrast, di- and oligosaccharides, gram-negative cell wall components, and electron donating reactions decreased significantly in the OP group (q<0.01) (Figure 7A). Then, we identified the relative bacteria through the ORF reads of those genes and found that *Ruminococcus*, *Helicobacter*, and *Lachnospiracea incertae sedis* might be potentially correlated with senile osteoporosis (Figure 7B–7D). To perform enrichment analysis, functional genes were also counted as log2 (fold change) values with significant differences (q<0.01), and changes in the abundance of functional genes were detected based on the Nr database. As shown in Figure 8, the ten genes with the most significant difference between the two groups were observed, including the species to which the genes belong. Overall, the genes could be classified into three categories: (1) synthesis and metabolism of carbohydrates: glucokinase, beta-glucosidase, beta-mannosidase, alpha-mannosidase, glycoside hydrolase; (2) genetic material activity: translation initiation factor IF-2, 2’ 3’-cyclic-nucleotide 2’-phosphodiesterase, DNA polymerase I; (3) protein and small molecular organics interaction: glycerol kinase, anion transporter, lipoprotein putative, aspartate 4-decarboxylase, outer membrane receptor protein, 3-dehydroquinate synthase, tRNA (adenosine(37)-N6)-threonylcarbamoyltransferase complex transferase subunit TsaD, aBC-type dipeptide/oligopeptide/nickel transport system ATPase component, bacteriolytic enzyme, TRAP transporter, and 4TM/12TM fusion protein. The glucose metabolism ability was relatively weakened in the OP group. According to the results of gene annotation, gene of glucokinase, anion transporter and translation initiation factor IF-2 had common gene sequences with *Alistipes* and *Helicobacter* which were marked in Figure 7. It was reported that up-regulation of anion transporter gene was associated with interleukin-17A (IL-17A) production [16]. So we speculated that the genes of function with significant differences from sequencing were correlated to *Alistipes* and *Helicobacter*.

## DISCUSSION

To the best of our knowledge, this is the first examination of specific changes in gut microbiota composition and function in an aged rat model of senile osteoporosis using both 16S rRNA and metagenomic sequencing. Higher OTUs numbers accompanied by lower diversity in gut microbiota were found in senile osteoporotic rats. Functional gene analyses indicated that alterations in the GM might contribute to senile osteoporosis through several metabolic pathways.

Different from clinical studies of GM [12, 14], we found that the OP group had a lower alpha diversity than the control by calculating four indexes. However, another clinical study showed a decreasing trend of alpha diversity in patients with osteoporosis compared with the normal bone mineral density (BMD) control [13], which is similar to our results. These discrepancies might potentially be due to the different numbers of included patients, gender compositions, diagnosis criteria of osteoporosis, population information (race, age), living habits including diet and exercise, and medicine use including antibiotics and anti-osteoporosis drugs in these clinical studies [17, 18]. Furthermore, the changing trend of OTUs numbers was inconsistent in these studies [12–14]. Thus, the roles of microbial diversity and OTUs remain a subject of debate and requires further study. The F/B ratio has been suggested to be another marked indicator of microbial structure determined by taxonomic composition [19]. In this study, a decreased F/B ratio caused by a reduction in Firmicutes and an increase in Bacteroidetes could be considered a variation in organisms. A previous report also showed a decreasing trend in the F/B ratio in a rat model of hypertension [20]. However, in studies of osteoporosis, only Li et al. considered that there was a decline in the F/B ratio of the Low-BMD group [12]; the other two studies both detected an increase in the F/B ratio in the osteoporosis group [13, 14]. Thus, the role of the F/B ratio as a marked indicator in osteoporosis requires further study.

To investigate taxonomic changes, we calculated the microbial abundance of the GM at the phylum level and observed the expansion of *Proteobacteria* from the phylum to species classification levels in the osteoporosis group. Recent studies have shown that the abundance of *Proteobacteria* can indicate unstable gut microbial communities and metabolic disorders [21, 22]. Then, we also calculated the abundance of the GM at the genus level. *Helicobacter* was the bacterium most positively related with osteoporosis and showed an increasing trend. In addition, *Helicobacter rodentium* is regarded as a potential pathogen that could cause increased immune-related gene expression after infection [23]. Thus, we speculated that the enrichment of *Helicobacter* was tightly related to osteoporosis as a risk factor. In addition, as the dominant bacterial group, *Firmicutes* plays a vital role in the global microbiota, and the dramatic changes in the abundance of *Ruminococcus* and *Lachnospiraceae* may be consistent with the variation in *Firmicutes*.

To detect how the GM is involved in the host by metabolism or biological activity, metagenomics was performed to analyze the functional characteristics of the main differential microbiota and global GM between the two groups. Genes of iron acquisition and metabolism and sulfatases and sulfatase modifying factor 1 (and a hypothetical) were more enriched in aged rats. Iron-sulfur clusters are crucial for mitochondrial metabolism and pulmonary hypertension; these processes are closely correlated with oxidative stress, and the related signaling pathway plays a major role in estrogen deficiency-induced osteoporosis [24, 25]. According to the taxonomy results annotated by ORF reads of functional genes, we found that *Helicobacter* matched well. The metabolism of *Helicobacter* includes glucose metabolism, amino acid metabolism, fatty acid and phospholipid metabolism, and the uptake and synthesis involved in the biosynthesis of nucleotides, nitrogen sources and iron uptake [26]. Therefore, *Helicobacter* was speculated to be the potential pathogenic factor of senile osteoporosis in both abundance and function. Lachnospiraceae was another differential species in our study; Lachnospiraceae mainly provides energy through carbohydrate metabolism in the intestine. Colonization of germ-free mice could induce a significant increase in blood glucose levels and mesenteric adipose tissue weights [27]. Furthermore, di- and oligosaccharide utilization showed a downward trend in osteoporotic rats, indicating that osteoporosis-associated glucose metabolic disturbance is characterized by a reduction in Lachnospiraceae. In recent years, it has been found that overweight, obesity and low body mass can be risk factors for osteoporosis and osteoporotic fracture [28].

The enrichment analysis in the current study based on the Nr database was mainly categorized into the synthesis and metabolism of carbohydrates, genetic material activity, protein and small molecular organic interactions. There is no doubt that aging results in changes in the GM, including composition and function; correspondingly, changes in the activity of genetic material could drive the GM to participate in the decline of immune system function and low-grade chronic inflammation [29]. Increasing glucokinase has been proven to be an adaptive mechanism after mandibular osteotomy and to enhance bone absorption in hypoxia [30]. Glucokinase transported from the gut to the circulatory system and bone may be involved in the bone remodeling balance. Thus, the comparison results of functional annotation based on both databases indicated that shifts in the GM contribute to aged osteoporosis through metabolic pathways and subsequent immune disorders.

It should be noted that the association of the gut microbiota and senile osteoporosis does not prove the causal relationship between the intestinal flora and the specialized disease: the physiological disorder caused by the disease can change the structure and function of the intestinal flora; on the contrary, intestinal microecological disorder may also lead to the occurrence of the disease. Fecal microbiota transplantation (FMT) is a main method to verify the direct evidence of gut microbiota dysbiosis in a specific disease [31]. In the future, studies based on FMT should be conducted to verify the causal relationship of gut microbiota dysbiosis on bone loss in senile osteoporosis. Probiotics and prebiotics have shown promising results in basic and clinical studies for the treatment of osteoporosis [18]. These methods, including FMT and medicines acting on the gut microbiota, would be promising for the prevention and treatment of senile osteoporosis.

According to the guidelines for primary osteoporosis, we used an aged rat model to illustrate the organismal and functional variations of the gut microbiota in senile osteoporosis [32]. The main population of senile osteoporosis is still female and to avoid the statistical deviation caused by gender, only female rats were selected in this study. Other scholars also proven that female rats could be used as the standard animal model of aged osteoporosis [33, 34]. However, there are still some limitations of this study, and further analysis is needed in the future. First, although beta diversity could cluster microbial communities well, the small sample size was still a deficiency of this study. Second, the intestinal flora of rats was not exactly the same as that of humans. Moreover, we only detected the genomic features of the GM by two sequencing methods, and transcriptomics and metabonomics were also needed to focus on the connection of the GM with the host in mechanism. Third, hormonal parameters (e.g., estrogen, parathyroid function), adipose tissue, liver kidneys, muscles, and parathyroid glands, which were not detected in this study, might help further detail the features of senile osteoporosis in rats. Therefore, further studies of the mechanism and verification are urgently required to clarify the role of GM in the pathogenesis of senile osteoporosis.

To summarize, this study reveals the association of gut microbiota composition and function with an aged rat model of senile osteoporosis in a brand new way, indicating that variations in the GM might contribute to senile osteoporosis through metabolic pathways. In addition, the notable microbial communities were significantly correlated with shifts in several special functions, indicating that senile osteoporosis was associated with dysbiosis of the GM in composition and relevant metabolic pathways. All the results potentially provide mechanistic evidence of the important role of the GM in senile osteoporosis progression.

## MATERIALS AND METHODS

### Study design

All animal experiments were approved by the Ethical Committee of Experimental Animal Care of the Shanghai Ninth People’s Hospital (SH9H-2019-A17-1). Conventional female SD rats of the control group (CON, 6 months old) and senile osteoporosis group (OP, 22 months old) were purchased from Slac Laboratory Animal Company (Slac., China, SCXK2012-0002). Rats in each group (n=4) were allowed free access to water and pelleted rodent diet and housed in two per cage with a 12-h light-dark cycle. After two weeks of acclimatization, bones and fecal samples of each rat were collected without any intervention. The body length and body weight of each rat were measured before sacrifice.

### Fecal microbiota sampling and microbial genomic DNA extraction

After one week of conventional raising, a sterile 15 ml tube was used to collect fecal samples from each rat directly (280 ± 20 mg) and stored at −80°C for subsequent sequencing. Microbial genomic DNA was extracted from fecal samples using a QIAamp DNA Stool Mini Kit (QIAGEN, USA).

### 16S rRNA and metagenome sequencing

The primers 338F and 806R were used to amplify the V3-4 region of 16S rRNA genes (TransStart Fastpfu DNA Polymerase). The thermocline conditions for amplification included 20 cycles (45 s at 95°C, 30 s at 55°C and 30 s at 72°C). The Illumina MiSeq instrument was used to perform pyrosequencing. To assemble the paired FASTQ files, Mothur (a software based on the principle of De Bruijn graph, version 1.39.5) was used [35].

The microbial DNA extracted from the samples for metagenomics sequencing was broken into 400 bp reads, and a DNA library was constructed using the NEXTflex™ DNA Sequencing Kit compatible with Biomek® FXp (Bio Scientific, USA). The paired-end reads were generated using an Illumina HiSeqTM 2500 after cluster generation. Raw FASTQ files were filtered using FASTX-Toolkit, and clean reads could be obtained for the following analysis. High-quality reads were spliced and assembled to obtain contigs by Mothur, according to the known sequence of contigs that can construct scaffolds. According to the total number of scaffold N50, scaffolds, and contigs and the length of other assembly effect indicators, we synthetically evaluated the best assembly results of Kmer.

### Bioinformatics

The high-quality DNA sequences were grouped into OTUs based on the SILVA reference database (V128) at 97% similarity [36]. Mothur was used to perform community richness and diversity analysis (Shannon, Simpson, ACE, Chao and Good’s coverage). To visualize the similarity or difference in data, principal coordinates analysis (PCoA) was performed by calculating the ecological distance between samples. The similarity of the community structure of each sample was calculated by the thetayc algorithm at the OTUs=0.03 level. The tree branch structure was displayed to describe the similarities and differences among multiple samples. Taxonomy was assigned by using the online software RDP classifier (80% threshold) based on the Ribosomal Database Project [37, 38]. Taxonomic characterization (phylum, class, order, family, genus, and species) was annotated by previous sequencing results using Magen software. A heat map diagram and clustering relationship diagram were constructed according to the classification information of OTUs. Redundancy analysis (RDA) was performed using the vegan package in R.

To calculate the read abundance of samples in the same group, genes were calculated with repetitive samples by SAMtools v0.1.19, and the MARS (MA-plot-based method with random sampling) model in the DEGseq program was used to detect the difference in expression abundance of each gene in the same groups. Diamond v0.9.1.102 was used to conduct BLASTP homology alignment between gene sequences and the databases (SEED database and Nr database) to obtain functional genes with statistically significant differences between the two groups, E value <10^-10^.

### Micro-CT analysis

After one week of conventional raising, all the animals were sacrificed; each tibia was collected in 4% paraformaldehyde and then scanned with MicroCT 81 (Scanco Medical AG, Bruettiselien, Switzerland). The spatial resolution of the system was 10 μm, the beam strength was set at 70 peak kV and 114 μA, and cubic voxels with a side length of 10 μm were used to represent the measured object. From the resulting voxel data, a volume of interest (VOI), 2-3 mm of the tibia trabecular region beginning 3 mm below the tibia plateau, was selected. The gray-value images were segmented using a low-pass filter to remove noise and a fixed threshold to extract the mineralized bone phase (sigma=1.2, support=2, threshold=180). Trabecular bone mineral content, bone volume fraction, thickness, spacing, and number values were computed by Scanco Medical software. 3D reconstruction was performed by the built-in software.

### Statistical analysis

The statistical significance of bone mass and biochemical indexes was calculated using one-way analysis of variance (ANOVA) in GraphPad Prism (GraphPad Software Inc., USA, version 6.0). All data are expressed as the mean values ± standard deviation (SD). Statistical significance was indicated as follows: *p < 0.05; #p < 0.01. Comparisons of abundance values for taxonomy and functional genes were analyzed using the Kruskal-Wallis H test with Benjamini-Hochberg FDR multiple test correction by STAMP version 2.1.3. Values with q<0.01 were considered statistically significant.

### Ethics approval

The study received ethics approval from the Ethics Committee of Shanghai Ninth People’s Hospital (SH9H-2019-A17-1).
